# P-1888. Quantifying the Potential Impact of Dalbavancin on Health Care Metrics Associated with Outpatient Parenteral Antibiotic Therapy Management of *Streptococcus* and *Staphylococcus* Infections

**DOI:** 10.1093/ofid/ofae631.2049

**Published:** 2025-01-29

**Authors:** Lucy X Li, Sean Anderson, Seth Judson, Caitlin Visek, Jessa Brenon, William F Wright, Sara C Keller

**Affiliations:** Johns Hopkins University, Baltimore, Maryland; Johns Hopkins University, Baltimore, Maryland; Johns Hopkins University, Baltimore, Maryland; Johns Hopkins University, Baltimore, Maryland; Johns Hopkins Home Care Group, Baltimore, Maryland; Johns Hopkins University, Baltimore, Maryland; Johns Hopkins University School of Medicine, Baltimore, MD

## Abstract

**Background:**

Socially vulnerable patients often complete parenteral antibiotic courses in healthcare facilities due to injection drug use (IDU) history or unstable housing situations, which leads to significant personal and health system costs and prolonged hospitalization risks. Our aim was to assess the potential impact of dalbavancin (DBV) utilization on length of stay (LOS) and adverse outcomes in this patient population.

**Methods:**

Retrospective study of patients ≥ 18 years old who were followed in the Johns Hopkins Health System Outpatient Parenteral Antibiotic Therapy (OPAT) program from 1/1/2021 to 6/30/2023 for *Streptococcus* and *Staphylococcus* bone/joint and endovascular infections (n = 900). Descriptive statistics were used to evaluate demographic, microbiologic, and clinical characteristics. Potential demand for DBV was quantified by estimating the impact of transitioning patients to DBV in terms of possible hospital days saved and OPAT-related complications avoided. Subanalyses were completed for patients with IDU history.

**Results:**

Median patient age was 58 years (IQR 44-68 years) and 501/900 (56%) were male. Most patients received vancomycin (30%) followed by cefazolin (24.4%) or anti-staphylococcal penicillin (24.0%), which required central venous catheter placement in 86.6% of patients. The most common indication for OPAT was osteomyelitis (54.8%) followed by endovascular infection. The median LOS after finalization of antibiotic recommendations was 5 days (IQR 3-10 days) with 44.3% requiring skilled nursing facility (SNF) placement. 43.7% of patients experienced an OPAT-related adverse event, almost half of which involved a return to the hospital. Among OPAT patients, 16.4% had an IDU history and 75.8% of them injected within the last 6 months. These patients had longer LOS (8 vs 5 days) after finalization of antibiotic recommendations and more OPAT-related adverse events compared to patients without IDU history (48.0% vs 43.7%).

**Conclusion:**

Patients, especially those with recent IDU, could benefit from DBV in lieu of parenteral antibiotics to complete prolonged treatment courses. Completion of deep-seeded gram-positive infection therapy with DBV vs OPAT has the potential to shorten hospital LOS and reduce the incidence of OPAT-associated adverse events.

**Disclosures:**

All Authors: No reported disclosures

